# Seasonal dynamics of meroplankton in a sub-Antarctic fjord (Southern Patagonia, Chile)

**DOI:** 10.1007/s00300-021-02823-6

**Published:** 2021-03-30

**Authors:** Santiago E. A. Pineda-Metz, Américo Montiel

**Affiliations:** 1 Alfred-Wegener-Institut Helmholtz-Zentrum für Polar- und Meeresforschung, D-27568 Bremerhaven, Germany; 2 Laboratorio de Ecología Funcional, Instituto de la Patagonia, Universidad de Magallanes, 6200000 Punta Arenas, Chile

**Keywords:** Benthic invertebrate larvae, Magellan strait, Proglacial fjord, Glacio-marine fjord, sub-polar, Zooplankton

## Abstract

Knowledge of seasonal dynamics and composition of meroplankton (larvae of benthic invertebrates) is rather limited for sub-Antarctic regions. We studied the seasonal dynamics of meroplankton in a sub-Antarctic proglacial basin (Gallegos Sound, Chile), by examining changes in the meroplankton community in relation to hydrographic variables along four sampling cruises between early winter 2010 and late winter 2011. The local meroplankton community was composed of 39 larval morphotypes distributed among 11 major taxa, being polychaetes the best represented (15 larvae morphotypes), and bivalve the most abundant. We found distinct seasonal differences in terms of meroplanktonic composition and abundance, with higher abundance and larval morphotype number during austral spring and late winter, and lower in summer and early winter. The pattern observed for meroplankton was directly related to seasonal variations of fluorescence of chlorophyll *a* and temperature. We found meroplankton abundances lower than those of other sub- and Polar environments. However, meroplanktonic temporal dynamics showed a common pattern for sub- and Polar fjords, suggesting a strong link between benthic spawning and the occurrence of phytoplankton blooms.

**Supplementary Information:**

The online version contains supplementary material available at 10.1007/s00300-021-02823-6.

## Introduction

Most of the benthic invertebrate fauna is composed of sedentary organisms with relatively low to no mobility and, thus, low dispersal capabilities. Consequently, some benthic species developed planktonic larvae to increase their dispersal capabilities (Peck et al. 2010a). Larvae released by benthic invertebrates are known as meroplankton and play a key role on benthic population dynamics and their geographic distribution (Becker et al. 2007). Adult reproduction and larval release are regulated by the distinct seasonal variability of primary production, water temperature, and salinity (Morgan et al. 2009; Kuklinski et al. 2013; Michelsen et al. 2017). Residence time of larvae in the water column can span from hours to years (Thorson 1950; Pearse et al. 1991; Basedow et al 2004; Bowden et al. 2009), and is also regulated by these hydrographic parameters in combination with substrate availability and predation pressure (Johnson and Brink 1998; Johnson and Shanks 2003; Landaeta et al. 2013).

In sub- and Polar waters, studies on seasonality of meroplankton are scarce. Early studies dealt with the importance of planktonic larvae as a reproductive strategy for high-latitude benthos, resulting in the development of Thorson’s Rule (e.g., Thorson 1950; Mileikovsky 1971), which was put to test by more recent studies and literature reviews (e.g., Gallardo and Penchaszadeh 2001; Pearse and Lockhart 2004; Marshal et al. 2012). However, only few studies dealt with seasonal dynamics of meroplanktonic communities. While for Arctic (Fetze and Arntz 2008; Kuklinski et al. 2013; Stübner et al. 2016; Michaelsen et al. 2017) and sub-Arctic waters (Silberberger et al. 2016), studies on seasonal dynamics of meroplankton have increased, comparable studies in Antarctic and sub-Antarctic waters are still scarce (e.g., Stanwell-Smith et al. 1999; Freire et al. 2006; Aguirre et al. 2012; Presta et al. 2020).

In the sub-Antarctic fjord and channel system of southern Patagonia, the main focus has been on spatial dynamics of the whole meroplanktonic assemblage (e.g., Thatje et al. 2003; Meerhoff et al. 2014) or for selected taxonomical groups such as mollusks (e.g., Campos and Diaz 2007), and decapods (e.g., Mujica and Villanueva 2003). These studies on spatial dynamics have shown meroplankton to be associated with chlorophyll *a* concentration, specific water basins, or changes in freshwater input (e.g., Hamame and Antezana 1999; Thatje et al. 2003; Meerhof et al. 2014). However, studies on seasonal dynamics of meroplankton and how these are related to hydrographic parameters are scarce (e.g., Lovrich 1999; Aguirre et al. 2012; Meerhof et al. 2014), and within the context of climate change, this is a gap that needs to be filled to understand how meroplankton might be affected in the near future.

One widespread habitat found at sub- and Polar environments are fjords with tidewater glaciers, known as glacio-marine or proglacial fjords. These habitats are highly sensitive to cryosphere–ocean interactions and climate warming (Syvitski et al. 1987; Kędra et al. 2010; Grange and Smith 2013) and due to their distinct terrigenous inputs (e.g., glacial ice, sediments, and meltwater runoff) might exhibit substantial environmental differences in comparison with adjacent basins and shelves (Grange and Smith 2013). High-latitude proglacial fjords have been heavily affected by raise of air temperatures, showing an increase of freshwater and sediment input (e.g., Peck et al. 2010b; Salcedo-Castro et al. 2015). The sub-Antarctic southern Chile is a region where proglacial fjords are a commonly found feature (Warren and Aniya 1999). Here, glacial retreat has been recorded since the mid-1900s, with significant frontal retreats between 1968 and 1975, and more recently during the year 2000 (Rignot et al. 2003; Rivera et al. 2007).

Glacier retreat in proglacial fjords has led to an increased interest in how glacier-driven cryosphere-ocean interactions affect biological dynamics. Despite the increased scientific attention, the study on seasonal dynamics of meroplankton in proglacial fjords in the southern hemisphere remains exclusive to Antarctic waters (e.g., Stanwell-Smith et al. 1999; Freire et al. 2006). In this context, we present the first study of the meroplankton community of a sub-Antarctic proglacial fjord adjacent to the Cordillera Darwin Icefield (southernmost Chile). Our main objectives were (i) to investigate the seasonal dynamics of the local meroplankton community and (ii) to identify the environmental drivers responsible for changes in meroplankton composition and abundance.

## Material and methods

### Study area

**Fig. 1:**
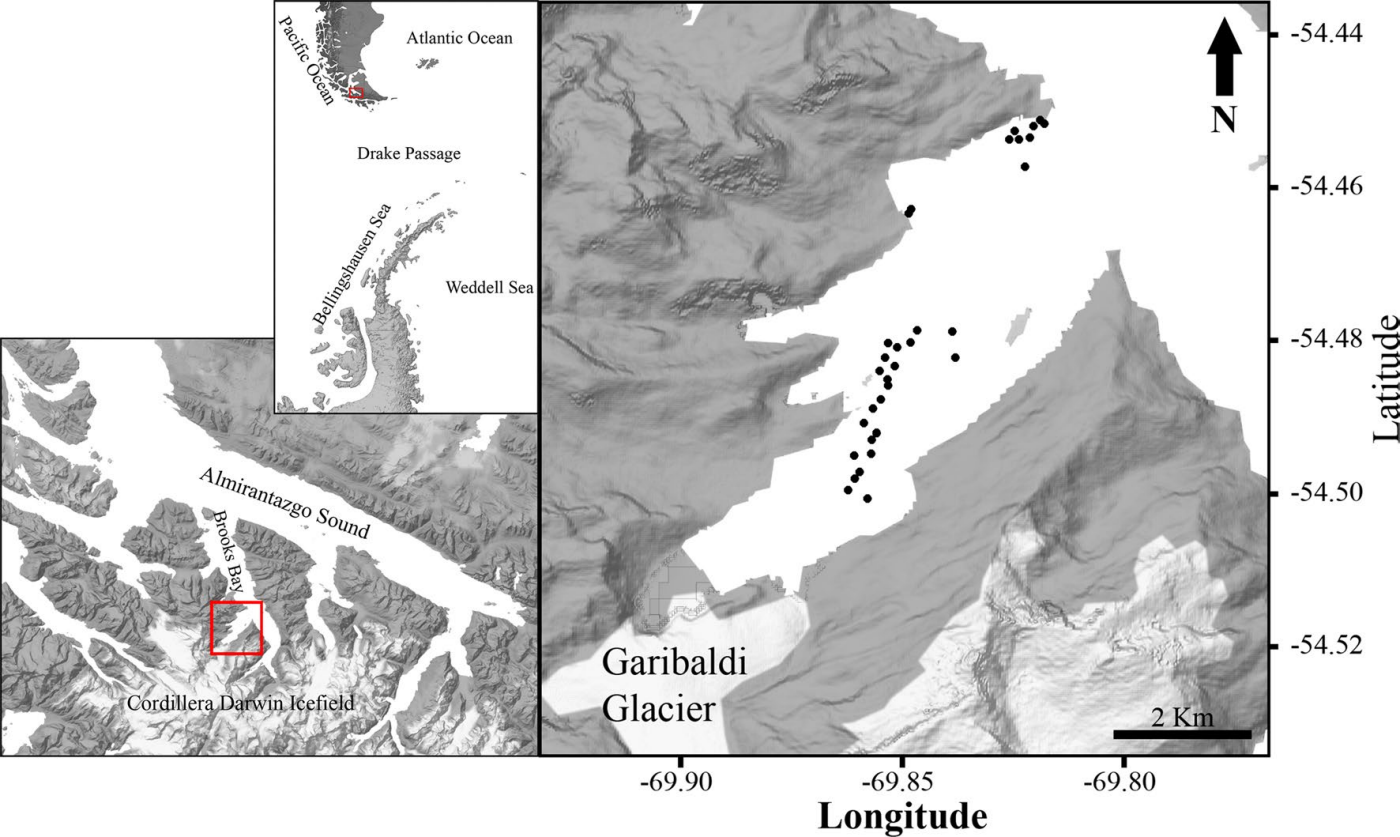
Locations of meroplankton stations sampled in the Gallegos Sound (Southern Patagonia)

Gallegos Sound is a 9-km long and 1.2- to 3.4-km-wide proglacial fjord adjacent to the Garibaldi glacier on the northeastern side of the Cordillera Darwin Icefield (Fig. 1). A sill is located within the fjord entrance at 50 m water depth dividing the fjord into two basins with maximum water depth of 100 and 170 m, respectively. The Garibaldi glacier is the main source of freshwater and sedimentary input into Gallegos Sound. During summer, ice floes can be observed floating in the proximity (up to a few km) of the glacier, whereas in winter, a thin sea ice layer (few cm thick) extends to up to 1 km from the glacier. Runoff material from the glacier, sea ice, ice floes, and freshwater streams flows from the fjord into Brookes Bay, which is connected to the Almirantazgo Sound (Fig. 1), a large multi-arm fjord system connected to the Magellan Strait.

The water column in the fjord is vertically stratified by a thermo- and halocline located at approximately 10 m water depth. The two layers were characterized by Salcedo-Castro et al. (2015) as an upper brackish water layer with relatively high primary production, low temperatures, and salinities, and a deeper layer with lower productivity which is slightly warmer and saltier. The brackish water layer has higher temperature variations (4.2 to 10.8 °C) and slightly lower salinity range (21.4 to 29.6) than the deeper layer (5.8–9.7 °C and 21.4–30.4, respectively). The water column in Gallegos Sound is well oxygenated throughout the year with oxygen concentrations > 8 mg L^−1^, and chlorophyll *a* concentrations ranging from >2 to >16 mg L^−1^. More details on the seasonality of hydrographic characteristics of the fjord from May 2010 to January 2011 are given by Salcedo-Castro et al. (2015).

### Meroplankton data

A total of 144 vertical tows were performed during four plankton sampling cruises between August 2010 and September 2011 (36 per campaign). The campaigns in 2010 correspond to early winter (August) and spring (November), whereas the 2011 campaigns represent summer (January) and late winter of 2011 (ends of September). The vertical tows represent three sections of a longitudinal transect from the glacial front to the mouth of the fjord, these sections being glacial front (GF), intermediate (IN) and mouth of the fjord (MF), and at each, twelve casts were done. Twelve tows were done at each section, half of them covered the upper 5 m of the water column and represent the brackish water layer, whereas the other half comprised the whole water column down to 5 m above seabed. To guarantee a more complete picture of the meroplankton community, we conducted our analysis considering the casts which covered the entire water column, i.e., 18 of the 36 casts per sampling campaign. Each sample was collected during daytime with a HYDROS-BIOS net with a 200 µm mesh size and 0.02 m^2^ opening area. All planktonic samples were fixed in a 5% formaldehyde seawater solution buffered with borax and stored in glass bottles for later analysis back in the laboratory.

All planktonic organisms in the samples were counted and determined under a stereomicroscope at 10x and 60x magnification, and the meroplankton larvae were separated from the remaining holoplanktonic organisms. After this separation, we excluded from our analyses four samples with meroplanktonic abundance = 0 (see Online Resource 1 for a complete list of stations), i.e., N = 72. Larvae were identified based on morphology and grouped into operational taxonomic units (OTU), using the identification guides of Palma and Kaiser (1993), Smith and Johnson (1996), Shanks (2001) and Young (2002). Abundance values were calculated in ind m^−3^ using the counted organisms and the volume of water that was sampled by the net. Due to the net not being equipped with a flowmeter, the volume sampled by the net in each tow was estimated using the equation $V={r}^{2}.h$, where *r* is the radius of the opening area of the net, and *h* represents the depth of the tow (modified after Palma and Kaiser 1993).

### Environmental data

Data for spring and early winter of 2010 and summer of 2011 were extracted from the study of Salcedo-Castro et al. (2015). For late winter of 2011, hydrographic data were measured with the same protocol used by Salcedo-Castro et al. (2015). At sixteen stations, a Seabird Electronics SBE19 plus CTDO was deployed to obtain vertical profiles of temperature, salinity, dissolved oxygen, and fluorescence of chlorophyll *a*. These CTD data were averaged for the whole water column.

Additional to CTD casts, discrete water samples were taken at 0, 5, 10, 20, and 30 m depth with a 5 L Niskin bottle. These water samples were used to calculate content of total suspended solids (TSS) by filtering seawater through a pre-weighted 0.45 µm nominal-pore polycarbonate filters and determining the weight difference after drying the sample at 60 °C for approximately 48 h. As for the CTD data, TSS values were averaged for the whole water column.

### Statistical analysis

Meroplanktonic abundance of each meroplankton cast was fourth root transformed to reduce the effects of highly dominant OTUs, and used to calculate a triangular matrix based on between-station Bray-Curtis similarities (Bray and Curtis 1957). The resemblance pattern given by the similarity matrix was visualized using a 2-d non-parametric multidimensional scaling (nMDS) plot. A complementary permutational multivariate analysis with 9999 permutations (PERMANOVA; Anderson 2001) was used to test for differences among and between sampling seasons. The Bonferroni correction (Bonferroni 1936) was used to adjust p values of the PERMANOVA analysis. Additionally, the indicator species analysis (ISA; De Cáceres and Legendre 2009) and similarity percentages test (SIMPER; Clarke 1993) were performed to discriminate characteristic OTUs for each season and contribution of OTUs to differences between seasons, respectively. Comparisons between longitudinal transects showed no significant differences between the sections GF, IN, and MF regardless of the season (PERMANOVA and post hoc corrected *p* > 0.05). Thus, we only focused on the temporal aspect of the sampling due to a lack of spatial gradients within Gallegos Sound.

Hydrographic data for each station were obtained from its closest CTD and Niskin bottle casts. Environmental data of all stations were arranged in a single matrix, which was used to test for co-correlation between environmental factors by means of the variance inflation factor (VIF; O’Brien 2007). Variables with a VIF > 10 were not further considered and were excluded in a step-by-step fashion. After this, we excluded water salinity, which correlated with water temperature. Hence, our final environmental matrix consisted of dissolved oxygen content, water temperature, fluorescence of chlorophyll *a*, and TSS.

To describe the relationship between seasonal changes in terms of environmental factors and meroplanktonic community, we fitted the environmental vectors onto the meroplanktonic nMDS ordination by using the envfit function of the vegan package for R (R Core Team 2019). Our meroplankton and environmental matrices were used in a redundancy analysis (RDA; Legendre and Anderson 1999) to describe the relationship between meroplanktonic abundance and hydrographic variables. To test the relation of environment and each OTU group, multiple linear regressions were done considering the variables included in the best RDA model. All statistical analyses and figures were done with the vegan, ggplot2, indicspecies, ade4, dplyr, ggmap, grid, ggsn, pairwise.adonis.r, and extrafont packages for R (R Core Team 2019).

## Results

**Fig. 2:**
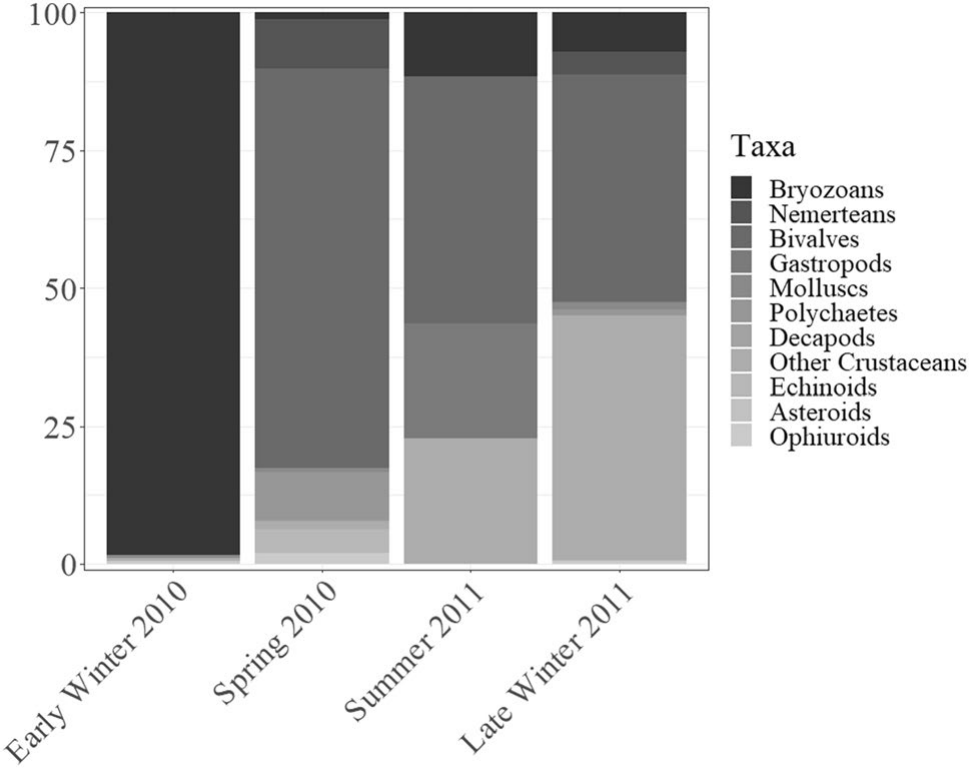
Taxonomic composition found for all season periods in the Gallegos Sound (Southern Patagonia)

A detailed list of the 39 identified larval OTUs can be found in Table. 1. These OTUs were distributed among 11 major taxa. Polychaetes dominated in terms of number of OTUs (15) but showed low abundances compared to taxa with 1to 3 OTUs, such as unidentified crustaceans, molluscs, or nemerteans. Overall, bivalve veliger 1 larvae were the most abundant, representing a high proportion of the meroplanktonic community between spring 2010 to late winter 2011 (Fig. 2 and Table 2), whereas bryozoan larvae (cyphonautes) clearly dominated in early winter 2010. The ISA showed a link between several meroplanktonic OTUs and periods where phytoplanktonic blooms start or are underway (Online Resource 2). Twenty one of all OTUs were significantly associated to one sampling season: 15 OTUs to late winter 2011, 5 to spring 2010, and 1 to summer 2011. Only 3 OTUs were significantly associate to more than one season. Cyphonautes was associated to early and late winter; whereas bivalve veliger 1 and echinopluteus 1 were associated to spring 2010 and late winter 2011 (Online Resource 2).

**Table 1:** Average abundance (ind m^−3^) and OTU data per taxon identified in the meroplanktonic samples collected during four sampling campaigns in Gallegos Sound (Southern Patagonia)

TAXA	OTU	2010		2011	
		Early winter (n = 18)	Spring (n = 18)	Summer (n = 14)	Late winter (n = 18)
Bryozoa					
	Cyphonautes	37 (± 9)	2 (± < 1)	1 (± < 1)	80 (±19)
Nemertea					
	Pilidium 1	0	7 (±2)	0	44 (±10)
	Pilidium 2	0	< 1 (± < 1)	0	3 (± < 1)
	Pilidium 3	0	6 (±1)	0	0
Bivalvia					
	Veliger 1	0	108 (±25)	4 (± 1)	468 (±110)
	Veliger 2	0	0	0	< 1 (± < 1)
Gastropoda					
	Veliger	0	0	2 (± < 1)	< 1 (± < 1)
Mollusca*					
	Trochophora 1	< 1 (± < 1)	< 1 (± < 1)	0	2 (± < 1)
	Trochophora 2	0	< 1 (± < 1)	0	2 (± < 1)
	Trochophora 3	< 1 (± < 1)	0	0	10 (±2)
Polychaeta					
	Trochophora 1	0	5 (±1)	0	< 1 (± < 1)
	Trochophora 2	0	< 1 (± < 1)	0	1 (± < 1)
	Trochophora 3	0	< 1 (± < 1)	0	< 1 (± < 1)
	Trochophora 4	0	2 (± < 1)	0	< 1 (± < 1)
	Trochophora 5	0	< 1 (± < 1)	0	3 (± < 1)
	Trochophora 6	0	0	0	< 1 (± < 1)
	Trochophora 7	0	< 1 (± < 1)	0	0
	Trochophora 8	0	0	0	< 1 (± < 1)
	Trochophora 9	0	< 1 (± < 1)	0	0
	Trochophora 10	0	0	0	4 (±1)
	Trochophora 11	0	0	0	< 1 (± < 1)
	Trochophora 12	0	0	0	< 1 (± < 1)
	Trochophora 13	0	0	0	< 1 (± < 1)
	Trochophora 14	0	0	0	< 1 (± < 1)
	Trochophora 15	0	4 (± < 1)	0	0
Decapoda					
	Zoea 1	0	< 1 (± < 1)	0	0
	Zoea 2	0	< 1 (± < 1)	0	1 (± < 1)
	Zoea 3	0	0	0	< 1 (± < 1)
Crustacea*					
	Nauplius 1	< 1 (± < 1)	2 (± < 1)	2 (± < 1)	489 (±115)
	Nauplius 2	0	< 1 (± < 1)	0	11 (±3)
Echinoidea					
	Echinopluteus 1	0	6 (±1)	0	1 (± < 1)
	Echinopluteus 2	0	< 1 (± < 1)	0	2 (± < 1)
Ophiuroidea					
	Ophiopluteus 1	1 (± < 1)	< 1 (± < 1)	0	5 (± 1)
	Ophiopluteus 2	0	2 (± < 1)	0	0
	Ophiopluteus 3	0	< 1 (± < 1)	0	< 1 (± < 1)
	Ophiopluteus 4	0	< 1 (± < 1)	0	0
	Ophiopluteus 5	0	< 1 (± < 1)	0	< 1 (± < 1)
	Ophiopluteus 6	0	0	0	< 1 (± < 1)
Asteroidea					
	Bipinnaria	0	0	0	< 1 (± < 1)
Total		37	148	10	1127

Values in brackets represent standard error

*Unidentified

**Table 2:** Summary of seasonal characteristics of meroplankton sampled in the Gallegos Sound (Southern Patagonia)

	2010		2011	
	Early winter	Spring	Summer	Late winter
Number of samples	18	18	14	18
Total abundance (ind m^-3^)	37	148	10	1127
Species number (S)	5	26	4	32
Dominant taxa (OTU)	Bryozoa (Cyphonautes)	Bivalvia (Veliger 1)	Bivalvia (Veliger 1)	Crustacea* (Nauplius 1)

*Unidentified

**Fig. 3:**
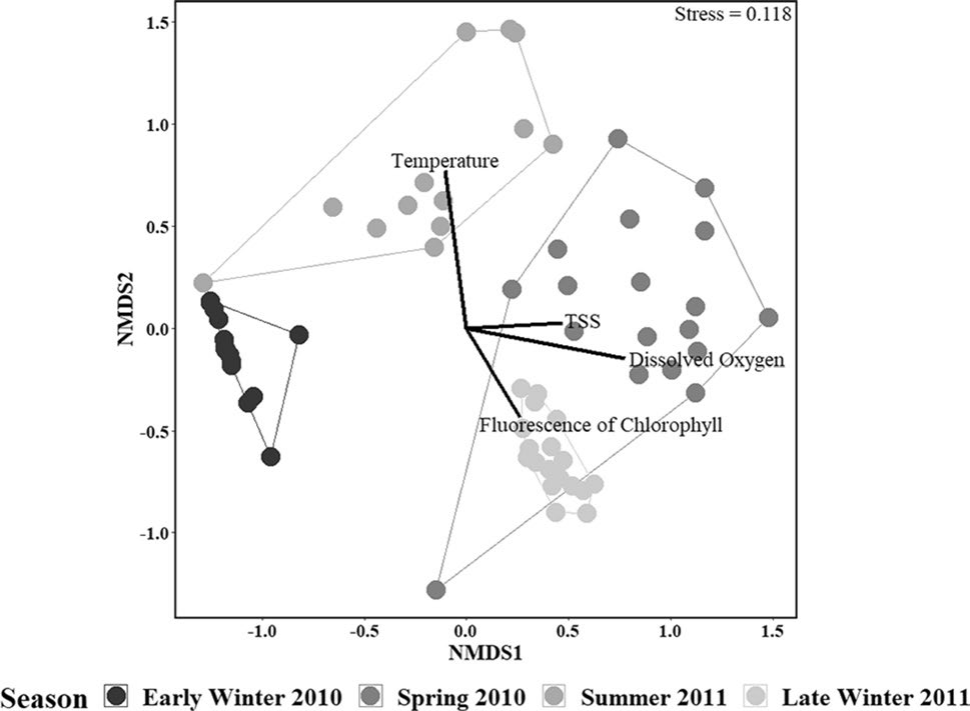
Two-dimensional non-parametric multidimensional scaling (nMDS) plot visualizing among-station resemblance pattern of meroplanktonic OTUs identified during the four sampling season periods in Gallegos Sound (Southern Patagonia). The pattern is based on between-station Bray-Curtis similarities calculated from abundance (ind m^-3^) data. Polygons represent the convex hull of each season. Black lines represent the fit of environmental vectors to the nMDS ordination

The grouping of stations shown by the nMDS (Fig. 3) suggests seasonal differences in terms of meroplanktonic abundance and composition. The PERMANOVA and its post hoc test showed these between-season differences to be significant (PERMANOVA corrected *p* < 0.001; see Table 3 and Online Resource 3 for pairwise comparison). A complementary SIMPER showed within dissimilarities among the four sampling seasons were low (18.32%) to intermediate (50.57%), whereas between-season dissimilarities were intermediate (66.88%) to high (92.14%; Table 3 and Online Resource 3). The SIMPER determined cyphonautes, bivalve veliger 1, and crustacean nauplii 1 larvae to contribute the most to between-season dissimilarities (Table 3). This can be observed in terms of meroplanktonic composition, which undergoes a first shifts from a cyphonautes dominated community in early winter 2010 to a bivalve veliger dominated one during spring 2010 and summer 2011, and a later second shift to a mixed bivalve veliger/crustacean nauplii dominated community in late winter 2011 (Fig. 2). The results of the enfit routine showed seasonal changes in meroplanktonic composition and abundance to be related to environmental seasonal variations (all *p* < 0.01), especially to dissolved oxygen which fitted best to the first axis of the nMDS (*r*^*2*^ = 0.509, *p* < 0.001; Fig. 3), and temperature which fitted best to the second axis of the nMDS (*r*^*2*^ = 0.598, *p* < 0.001; Fig. 3).

**Table 3:** Within- and between-group dissimilarities based on composition and abundance of meroplanktonic OTUs found for early winter and spring 2010, and summer and late winter 2011 in Gallegos Sound (Southern Patagonia). Within- and between-group dissimilarities are given in percentage

Year	Season	2010		2011	
		Early winter (n = 18)	Spring (n = 18)	Summer (n = 14)	Late winter (n = 18)
2010	Early winter	**18.32**			
	Spring	92.14* Bivalvia Veliger 1 (20.60) Cyphonautes (13.20)	**50.57**		
2011	Summer	77.18* Cyphonautes (30.94) Bivalvia Veliger 1 (22.64) Nauplius 1 (10.83)	76.49* Bivalvia Veliger 1 (12.30) Polychaeta Trochophora 1 (6.66) Pilidium 3 (6.54)	**45.30**	
	Late winter	83.05* Bivalvia Veliger 1 (14.67) Nauplius 1 (14.16) Pilidium 1 (8.08)	66.88* Nauplius 1 (9.00) Cyphonautes (5.48) Mollusca Trocophora 3 (4.05)	80.76* Nauplius 1 (11.43) Bivalvia Veliger 1 (9.82) Pilidium 1 (7.86)	**31.07**

Values in brackets correspond to the contribution (in percentage) of each OTU to between-group dissimilarities. The top three OTUs contributing to between-group dissimilarities are given (see Supplementary Table 3 for an extended list)

*Significantly different at p (adjusted)<0.006

**Fig. 4:**
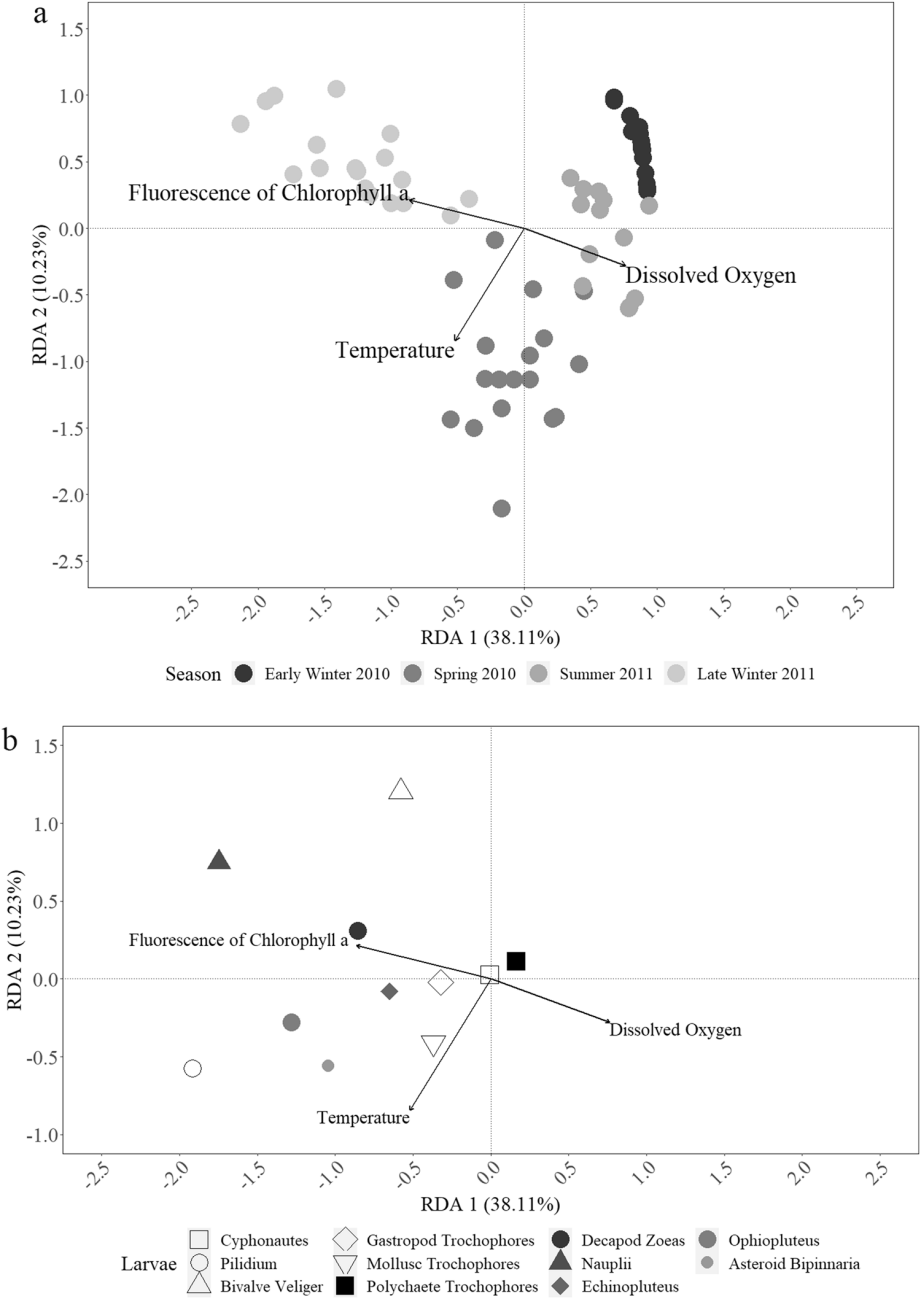
Redundancy analysis (RDA) on fourth root transformed meroplanktonic larval group data showing the ordination with a) stations and environmental variables and b) larval groups and environmental variables. The eigenvalues as percentages are provided for the first two RDA axis with a total of 48.34% of variance explained

Our RDA showed significant relationships between meroplanktonic abundance with environmental gradients, which explained 48.34% of the meroplanktonic variability (adjusted *r*^*2*^ = 0.482, *p* < 0.001). An ANOVA-like permutation test showed only temperature, fluorescence of chlorophyll *a*, and dissolved oxygen to significantly contribute to the ordinations based on abundance of OTU groups (*p* < 0.001, Permutations = 9999; Fig. 4a and b). The first RDA axis explained 38.11% of the meroplanktonic variability and was mainly related to fluorescence of chlorophyll *a*, whereas the second explained 10.23% of the variability and was mainly related to temperature (Fig. 4a and b).

The RDA results (Fig. 4a and b) suggest meroplanktonic seasonal differences to be associated to seasonal changes of fluorescence of chlorophyll *a* and temperature. The fluorescence of chlorophyll *a* gradient observed in the station-based RDA plot (Fig. 4a) suggests the phytoplankton bloom to occur between late winter and spring. The abundance and number of meroplanktonic OTUs appears to match the phytoplanktonic bloom, with high abundance and number of OTUs during spring 2010 and late winter 2011, and lower during early winter 2010 and summer 2011 (Table 2 and Fig. 2). The pattern observed based on temperature (Fig. 4a) reflects the temperature increment typical for spring and summer.

Individual multiple regression using the RDA model showed abundance of most OTU groups to be significantly related by the environmental variation (*p* < 0.005), only asteroid larvae abundance was independent of all environmental factors (*p* > 0.05). Both RDA and multiple regression results suggest meroplanktonic groups to be positively related to fluorescence of chlorophyll *a*, with the exception of polychaete trochophores and asteroid bipinnaria. The RDA suggest the spawning period of many benthic organisms to match with the phytoplanktonic bloom. Contrastingly, most larvae appear inversely related to dissolved oxygen (Fig.4b). We found temperature to also influence meroplanktonic abundance, albeit not as strongly as fluorescence of chlorophyll *a* and dissolver oxygen. Groups such as bryozoan cyphonautes, bivalve veliger, polychaete trochophores, decapod zoea, and crustacean nauplii larvae showed an inverse relation to temperature. Salinity was inversely correlated to temperature (Spearman statistic = − 0.603; *p* <0.001; Permutations = 9999), which suggests the abundance of these larval groups to be related to higher salinity values. Based in our results, the meroplanktonic groups of Gallegos Sound can be divided into three clusters: a) lower temperature-higher fluorescence of chlorophyll *a*, consisting of bryozoan cyphonautes, bivalve veliger, decapod zoea, and crustacean nauplii larvae; a b) higher temperature-higher fluorescence of chlorophyll *a* group composed of nemertean pilidium, mollusc trochophores, gastropod trochophores, unidentified mollusc trochophores, echinopluteus, ophiopluteus, and asteroid bipinnaria larvae; and c) a lower temperature-lower fluorescence of chlorophyll *a* composed only by polychaete trochophores larvae.

## Discussion

Our results comprise one of the first attempts to describe the temporal variation of the meroplanktonic assemblages and its relation to environmental gradients in a sub-Antarctic proglacial fjord of the southern Patagonia fjord and channel region. A region where knowledge on benthic reproductive biology and larval taxonomy and development history is scarce, especially for areas within the Strait of Magellan. The environmental seasonality is reflected in abundance and composition differences between samplings. Furthermore, we found several meroplanktonic larvae to be significantly related to a single sampling season. These results suggest benthos to spawn larvae at a specific season, most likely following an environmental trigger such as rise of temperatures or start of the phytoplankton bloom.

The amount of OTUs we identified in Gallegos Sound (n = 39) is within the described OTU range for meroplankton communities in Patagonian fjords and channels, where up to 53 OTUs were described (e.g., Thatje et al. 2003; Aguirre et al. 2012; Meerhoff et al. 2014; Presta et al. 2020). For most taxa, only one OTU per taxon was described (e.g., Deffren-Janson et al. 1999; Fernandez-Severini and Hoffmeyer 2005; Aguirre et al. 2012; Presta et al. 2020), whereas 4 to 44 larval types have been described for crustaceans (three, in our study). With the exception of bryozoan, gastropod, and asteroid larvae, most of the major taxonomic groups were represented by two or more larval OTUs, being polychaete the extreme case with 15 OTUs. On the one hand side, this might evidence a high frequency of taxonomical approaches to identify crustacean larvae and the special focus given to this group, which has a high economic importance in this region. On the other, our findings suggest that by identifying larvae using an OTU designation, results might show meroplanktonic diversity in the Patagonian fjord and channel region to be higher than previously thought.

For comparison purposes, we focused on austral late winter and spring because most meroplanktonic studies were carried out during these seasons. In Gallegos Sound, late winter and spring are seasons with high abundance of meroplankton where bivalve larvae clearly dominate in terms of abundance and polychaete larvae in terms of OTU richness. Our abundance values were lower than those obtained with similar mesh sizes for the Strait of Magellan and Beagle Channel (Thatje et al. 2003; Aguirre et al. 2012; Presta et al. 2020). Furthermore, the meroplanktonic composition in Gallegos Sound also differed from that described for the Strait of Magellan, where polychaete larvae dominated in terms of abundance (Deffren-Janson et al. 1999; Thatje et al. 2003). Contrastingly, the meroplanktonic composition for late winter and spring in Gallegos Sound was similar to that described for the Beagle Channel, i.e., a crustacean/bivalve-dominated larval community. For the Beagle Channel, Fernández-Severini and Hoffmeyer (2005), Aguirre et al. (2012), and Presta et al. (2020) described a community dominated by crustacean (represented by cirripedes) and bivalves. One reason for differences in terms of meroplanktonic characteristics might be the mesh size used in our sampling design (200 µm) which while commonly used by studies in the Strait of Magellan and Beagle Channel, it can result in underestimation of small size larvae and early developmental stages of larger larvae. Other reasons for these differences could be related to reproductive (e.g., timing of larval release) and development (e.g., larval residence time) factors, as well as due to differences in the benthic community found at the different sampling regions.

Different meroplankton composition and abundance between locations/regions might be related to local benthic abundance differences. Benthos in Gallegos Sound (A. Montiel unpublished data) also shows lower abundance than in other sites of the Strait of Magellan (Gerdes and Montiel 1999; Montiel et al. 2001; Montiel et al. 2011). Another factor is the proportion of benthic species which reproduce via larvae. Following Thorson’s rule (Mileikovsky 1971), the proportion of benthic spawning species should be relatively low, especially for gastropod molluscs and echinoids (Clarke 1992; Marshall et al. 2012). Based on our results, the benthos of Gallegos Sound should be composed of three to six mollusc species and 15 polychaete species. Benthic sampling done in parallel to our study suggests at least 26 mollusc and 46 polychaete species to be present in Gallegos Sound (A. Montiel unpublished data). This would imply that 11–23% of the mollusc and ~32% of the polychaete species reproduce via planktonic larvae, pending to be proved by a sampling strategy which considers a higher frequency of samplings. Thus, the combination of low proportion of spawning benthic species and low benthic abundances would explain the lower meroplanktonic abundance and OTU amount in Gallegos Sound as compared to other locations of the Strait of Magellan and Beagle Channel (Thatje et al. 2003; Aguirre et al. 2012; Presta et al. 2020), and also other sub- and Antarctic embayments (Shreeve and Peck 1995; Stanwell–Smith et al. 1999; Freire et al. 2006; Bowden et al. 2009).

Meroplanktonic larvae are linked to their benthic parents by means of pelago-benthic processes (Schnack-Schiel and Isla 2005, Pineda-Metz 2020). Thus, the composition of meroplankton should reflect the local benthos community (Michelsen et al. 2017). Conversely, the meroplanktonic composition regulates and maintains the local benthos community (Stanwell-Smith and Barnes 1997; Levin 2006). The meroplankton community in Gallegos Sound was dominated by cyphonautes (bryozoan) larvae during early winter, crustacean nauplii and bivalve veliger during late winter, and exclusively by bivalve veliger during spring and summer. This, however, is not reflected in the benthic community in Gallegos Sound (A. Montiel unpublished data), which appears similar to that of the Strait of Magellan (Gerdes and Montiel 1999; Montiel et al. 2001; Thatje and Brown 2009; Montiel et al. 2011) where polychaetes dominate followed by bivalves. However, these descriptions are based on soft-sediment samples and disregard hard-bottom communities, such as those found along the rocky walls in fjords and channels. Thus, we suggest cyphonautes and nauplii to originate from benthos inhabiting the walls of the fjord and/or to be import from adjacent basins. Polychaete larvae were represented by a higher number of OTUs as any other group (15 OTUs), which mirrors how polychaetes represent the benthic group with highest species number (> 40 species; A. Montiel unpublished data). However, polychaete larvae were a minor component of Gallegos Sound’s meroplanktonic abundance throughout our study period. This would suggest larval residence time to be relatively short (hours to days), spawning of larvae to occur at another point in time (e.g., late summer or autumn), or polychaete larvae to develop in adjacent basins and then return to its origin, in a similar fashion as the findings of Dittel and Epifanio (1982) for crab larvae.

We found a distinct seasonality in the meroplankton community with higher abundance and number of larval OTUs tightly related to seasonal temperature and dissolved oxygen differences, which matched to periods of high fluorescence of chlorophyll *a*, a proxy for primary production. We also distinguished larval groups inversely related to temperature, suggesting a link to higher salinities. This would imply some benthic organisms such as bryozoans, bivalves, crustaceans (including decapods), and polychaetes to also respond to salinity changes and, in general, with the phytoplanktonic bloom. Polychaetes appear to be the exception since their larvae were associated with low fluorescence and temperature (i.e., higher salinities), suggesting the spawning season to occur mid-winter, just before the phytoplankton bloom starts. In high latitudes, benthos shows a discontinuous spawning behavior, resulting in highly heterogeneous meroplankton dynamics (Picket 1980; Pearse et al. 1991; Thatje 2003). However, for sub- and Polar fjords, the observed pattern in Gallegos Sound appears to be common as similar results have also been reported for fjords in the Beagle Channel (Aguirre et al. 2012; Presta et al. 2020), the Baker and Martinez fjords in Southern Patagonia (Meereshoff et al. 2014), the Porsanger fjord in the Barents Sea (Michelsen et al. 2017), and in Admiralty Bay in King Georg Island (Freire et al. 2006). Our findings and those of other studies suggest high-latitude benthos living in fjords and closed bays to synchronize their spawning season to match the rise in temperatures during spring which is accompanied by local phytoplankton bloom, most likely to ensure the survivability of larvae by the presence of a high and rich supply of food for the released larvae.

In conclusion, the meroplanktonic community of Gallegos Sound presents strong seasonal dynamics similar to that described for other high-latitude fjords. This seasonality appears to be mainly driven by variations of fluorescence of chlorophyll *a* and temperature. Despite the diverse composition in terms of number of OTUs, the meroplanktonic community present in Gallegos Sound is less abundant in comparison to other areas of the Strait of Magellan, Beagle Channel, and sub- and Antarctic embayments. Furthermore, our results showed differences in terms of composition with those of meroplanktonic studies in the Strait of Magellan. We propose these differences to be due to contrasting local benthic characteristics, larval developmental times, and larval transport mechanisms.

## Supplementary Information

Below is the link to the electronic supplementary material.

Supplementary file1 (PDF 191 KB)

Supplementary file2 (PDF 366 KB)

Supplementary file3 (PDF 105 KB)

## Data Availability

Meroplanktonic and hydrographic data supporting this study will be made available in the PANGAEA data repository. Data can be provided upon request to the corresponding author.
