# Supplementary material for: Seasonal dynamics of meroplankton in a sub-Antarctic fjord (Southern Patagonia, Chile)
Source: Polar Biol. 2021 Mar 30;44(5):875–86. doi: 10.1007/s00300-021-02823-6 (PMC8008332; doi:10.1007/s00300-021-02823-6)
Supplement: Supplementary file 1 — Supplementary file1 (PDF 191 KB) [file 300_2021_2823_MOESM1_ESM.pdf]

Online Resource 1: List of station per sampling campaign considered in our study.

Cast Name Code	Latitude (-S, +N)	Longitude (-W, +E)	Sampling date	Season	Max Depth (m)
1	-54,4908667	-69,8590833	15-Aug-2010	Early Winter	20
2	-54,4908667	-69,8590833			
3	-54,48775	-69,8553667			40
4	-54,48775	-69,8553667			
5	-54,48595	-69,8536333			80
6	-54,48595	-69,8536333			
7	-54,4833667	-69,8522	16-Aug-2010		20
8	-54,4833667	-69,8522			
9	-54,4787167	-69,84705			40
10	-54,4787167	-69,84705			
11	-54,4803	-69,8485833			60
12	-54,4803	-69,8485833			
13	-54,4572833	-69,8228667	17-Aug-2010		20
14	-54,4572833	-69,8228667			
15	-54,4572833	-69,8228667			40
16	-54,4572833	-69,8228667			
17	-54,4534667	-69,82175			60
18	-54,4534667	-69,82175			
19	-54,4889333	-69,8570667	06-Nov-2010	Spring	20
20	-54,4889333	-69,8570667			
21	-54,4971833	-69,8601167			43
22	-54,4971833	-69,8601167			
23	-54,4950167	-69,86135			65
24	-54,4950167	-69,86135			
25	-54,4833833	-69,8521167	07-Nov-2010		28
26	-54,4833833	-69,8521167			
27	-54,4822833	-69,8385167			40
28	-54,4822833	-69,8385167			
29	-54,47885	-69,83925			60
30	-54,47885	-69,83925			
31	-54,4633667	-69,8490833	08-Nov-2010		20
32	-54,4633667	-69,8490833			
33	-54,4628	-69,8484667			40
34	-54,4628	-69,8484667			
35	-54,4537333	-69,8242			68
36	-54,4537333	-69,8242			
37	-54,49955	-69,8627167	26-Jan-2011	Summer	20
38	-54,49955	-69,8627167			
39	-54,4980333	-69,8612			40
40	-54,4980333	-69,8612			
41	-54,4948	-69,8575333			60

42	-54,4948	-69,8575333			
43	-54,49955	-69,8627167	27-Jan-2011		20
44*	-54.49955	-69.8627167			
45	-54,4851333	-69,8538667			40
46	-54,4851333	-69,8538667			
47	-54,4803667	-69,8536333			60
48	-54,4803667	-69,8536333			
49	-54,45195	-69,8208333	28-Jan-2011		20
50	-54,45195	-69,8208333			
51	-54,4516333	-69,8184667			40
52*	-54.4516333	-69.8184667			
53*	-54.4519	-69,8194833			60
54*	-54.4519	-69,8194833			
55	-54,4921333	-69,8562	16-Sep-2011		20
56	-54,4921333	-69,8562			
57	-54,49295	-69,8573333			40
58	-54,49295	-69,8573333			
59	-54,5006667	-69,8582833			60
60	-54,5006667	-69,8582833			
61	-54,484	-69,8556333			20
62	-54,484	-69,8556333			
63	-54,48225	-69,8542833		Late Winter	40
64	-54,48225	-69,8542833			
65	-54,48095	-69,8516667			58
66	-54,48095	-69,8516667			
67	-54,45115	-69,8193833	17-Sep-2011		23
68	-54,45115	-69,8193833			
69	-54,4525667	-69,82515			38
70	-54,4525667	-69,82515			
71	-54,4537167	-69,8263			60
72	-54,4537167	-69,8263			

*Stations with meroplanktnic abundance = 0
