# Supplementary material for: Seasonal dynamics of meroplankton in a sub-Antarctic fjord (Southern Patagonia, Chile)
Source: Polar Biol. 2021 Mar 30;44(5):875–86. doi: 10.1007/s00300-021-02823-6 (PMC8008332; doi:10.1007/s00300-021-02823-6)
Supplement: Supplementary file 2 — Supplementary file2 (PDF 366 KB) [file 300_2021_2823_MOESM2_ESM.pdf]

Online Resource 2: Within- and between-group dissimilarities based on composition and abundance of meroplanktonic OTUs found for early winter and spring 2010, and summer and late winter 2011 in Gallegos Sound (Southern Patagonia). Value in brackets correspond to the contribution of each OUT to between-group dissimilarities. Listed OTUs contribute to up to 50% of the between-group dissimilarities.

Year		2010		2011	
	Season	Early Winter (n = 18)	Spring (n = 18)	Summer (n = 14)	Late Winter (n = 18)
2010	Early Winter	**18.32**			
	Spring	**92.14*** Bivalvia Veliger 1 (20.60) Cyphonautes (13.20) Polychaeta Trochophora 1 (6.96) Pilidium 3 (6.84) Pilidium 1 (6.47)	**50.57**		
2011	Summer	**77.18*** Cyphonautes (30.94) Bivalvia Veliger 1 (22.64)	**76.49*** Bivalvia Veliger 1 (12.30) Polychaeta Trochophora 1 (6.66) Pilidium 3 (6.54) Pilidium 1 (6.19) Echinopluteus 1 (5.75) Polychaeta Trochophora 18 (5.42) Nauplius 1 (5.02) Cyphonautes (4.68)	**45.30**	
	Late Winter	**83.05*** Bivalvia Veliger 1 (14.67) Nauplius 1 (14.16) Pilidium 1 (8.08)	**66.88*** Nauplius 1 (9.00) Cyphonautes (5.48) Mollusca Trocophora 3 (4.05)	**80.76*** Nauplius 1 (11.43) Bivalvia Veliger 1 (9.82) Pilidium 1 (7.86)	**31.07**

	Mollusca Trochophora 3 (5.23)<br>Nauplius 2 (4.72)<br>Ophiopluteus 1 (3.66)	Bivalvia Trochophora 1 (3.97)<br>Pilidium 1 (3.58)<br>Nauplius 2 (3.43)<br>Ophiopluteus 1 (2.86)<br>Pilidium 3 (2.76)<br>Polychaeta Trochophora 10 (2.55)<br>Pilidium 2 (2.38)<br>Polychaeta Trochophora 18 (2.31)<br>Polychaeta Trochophora 1 (2.31)<br>Echinopluteus 1 (2.06)<br>Mollusca Trochophora 2 (2.05)<br>Polychaeta Trochophora 5 (1.78)	Cyphonautes (6.75)<br>Mollusca Trochophora 3 (5.26)<br>Nauplius 2 (4.59)<br>Ophiopluteus 1 (3.78)<br>Polychaeta Trochophora 10 (3.34)	

*Significantly different at p (adjusted)<0.006
