# Supplementary material for: Seasonal dynamics of meroplankton in a sub-Antarctic fjord (Southern Patagonia, Chile)
Source: Polar Biol. 2021 Mar 30;44(5):875–86. doi: 10.1007/s00300-021-02823-6 (PMC8008332; doi:10.1007/s00300-021-02823-6)
Supplement: Supplementary file 3 — Supplementary file3 (PDF 105 KB) [file 300_2021_2823_MOESM3_ESM.pdf]

Online Resource 3: Results of the Indicator Species Analysis (ISA). List of OTUs associated to a particular season or combination of them. Values in brackets represent the association of a given OUT based on a correlation statistic. All correlations are significant at $p < 0.005$

Season	Spring 2010	Summer 2011	Late Winter 2011	Early Winter 2010 + Late Winter 2011	Spring 2010 + Late Winter 2011
OTU	Pilidium 3 (0.790)<br>Polychaeta Trochophora 18 (0.751)<br>Polychaeta Trochophora 1 (0.672)<br>Ophiopluteus 2 (0.557)<br>Polychaeta Trochophora 3 (0.404)	Gastropoda Veliger (0.538)	Mollusca Trochophora 3 (0.948)<br>Nauplius 1 (0.904)<br>Nauplius 2 (0.854)<br>Ophiopluteus 1 (0.803)<br>Pilidium 1 (0.797)<br>Polychaeta Trochophora 10 (0.778)<br>Pilidium 2 (0.759)<br>Mollusca Trochophora 2 (0.605)<br>Polychaeta Trochophora 5 (0.597)<br>Mollusca Trochophora 1 (0.575)<br>Polychaeta Trochophora 2 (0.523)<br>Polychaeta Trochophora 11 (0.517)<br>Polychaeta Trochophora 13 (0.517)<br>Polychaeta Trochophora 8 (0.509)<br>Zoea 2 (0.475)<br>Echinopluteus (0.423)	Cyphonautes (0.846)	Bivalvia Veliger 1 (0.815)<br>Echinopluteus 1 (0.605)
